# Florida neighborhood analysis of social determinants and their relationship to life expectancy

**DOI:** 10.1186/s12889-020-08754-x

**Published:** 2020-05-06

**Authors:** Bertram L. Melix, Christopher K. Uejio, Kristina W. Kintziger, Keshia Reid, Chris Duclos, Melissa M. Jordan, Tisha Holmes, Jessica Joiner

**Affiliations:** 1 Department of Geography, Florida State University, Tallahassee, FL USA; 2 Department of Public Health, University of Tennessee, Knoxville, TN USA; 3 Division of Community Health Promotion, Tallahassee, FL USA; 4 Division of Disease Control and Health Protection, Tallahassee, FL USA; 5 Public Health Research Unit, Florida Department of Health, Tallahassee, FL USA; 6 Department of Urban and Regional Planning, Florida State University, Tallahassee, FL USA

**Keywords:** Life expectancy, Social determinants of Health, Social vulnerability, Spatial modeling, Spatial statistics

## Abstract

**Background:**

Social determinants of health (SDOH) contribute to unequal life expectancy (LE). Only a handful of papers have analyzed these relationships at the neighborhood level as opposed to the county level. This study draws on both the SDOH and social vulnerability literature to identify relevant factors affecting LE.

**Methods:**

LE was calculated from mortality records for Florida from 2009 to 2013 for 3640 census tracts with reliable estimates. A spatial Durbin error model (SDEM) quantified the direction and magnitude of the factors to LE. The SDEM contains a spatial error term and jointly estimates both local and neighborhood associations. This methodology controls for non-independence between census tracts to provide unbiased statistical estimates.

**Results:**

Factors significantly related to an increase in LE, include percentage (%) of the population who identify as Hispanic (beta coefficient [β]: 0.06, *p*-value [P] < 0.001) and % of age dependent populations (% population < 5 years old and % population > 65) (β: 0.13, *P* < 0.001). Conversely, the following factors exhibited significant negative LE associations, % of households with no automobile (β: -0.05, *P* < 0.001), % of mobile homes (β: -0.02, *P* < 0.001), and % of female headed households (β: -0.11, *P* < 0.001).

**Conclusions:**

Results from the SDEM demonstrate social vulnerability indicators account for additional geographic LE variability beyond commonly studied SDOH. Empirical findings from this analysis can help local health departments identify drivers of spatial health disparities at the local level.

## Highlights

- Life expectancy, or the average remaining time a person is expected to live, differs throughout local communities in Florida.
- Differences in life expectancy between neighborhoods, can in part, be attributed to the social determinants of health, or local conditions where people work, live, and age.
- This study draws on a social vulnerability dataset detailing factors meant to indicate which population groups are at risk to be harmed by natural and/or human caused disasters in Florida to more precisely characterize differences in life expectancy.

## Background

Life expectancy (LE) is defined as the number of years an average individual can expect to live given existing age-specific death rates [1]. This indicator is easily understood by policy makers and the general public and allows for direct comparisons across space and time. LE is a particularly useful metric for examining health disparities. The most important factors affecting LE vary geographically and are driven by a complex interplay of environmental, socioeconomic, political, and structural conditions [2]. SDOH embody the consequences of multifaceted societal processes and norms that shape living conditions and produce a broad range of health disparities [2–5]. Traditionally, public health studies use a social determinants of health (SDOH) framework to identify the most important factors affecting LE [2–5].

Multiple studies have examined the effects of SDOH on LE or mortality rates at the county or state level to unveil disparate health outcomes and identify areas where public health interventions might be necessary [6–9]. To operationalize SDOH, studies examine sociodemographic characteristics such as income and wealth, education, occupation, and racial or ethnic identity. Much of the variation among counties can be explained by the interplay of socio-economic, race/ethnicity, behavioral and health status, and health care factors [7]. However, county-level estimates may mask important differences across and between neighborhoods that comprise the county. At the county spatial scale, notable health differences between neighborhoods can be obscured. We define neighborhoods as census tracts, since they are more precise and completely contained within counties. Analyzing geographic neighborhoods often requires spatial methods that control for potential non-independence between adjacent census tracts. This study uses a spatial Durbin error model (SDEM) to investigate the impact of neighborhood SDOH on LE while controlling for unexplained spatial autocorrelation.

Recent studies have analyzed county level SDOH and LE [8, 9]. This study examines both SDOH and social vulnerability indicators to provide a multifaceted analysis of health disparities. Social vulnerability to environmental hazards refers to the ability for individuals at risk to be harmed by natural and/or human caused disasters to adapt or recover [10]. The SDOH indicators focus on broad metrics (e.g. income) that likely capture multiple societal processes (e.g. health insurance, education, the healthy worker effect) that produce disparities. By comparison, social vulnerability examines both broad and detailed risk indicators such as access to a car, living in a mobile home, persons per household, receiving social security benefits, and female headed households. Although less commonly used in SDOH studies, the SOVI index is popular among vulnerability researchers [11–13].

Key factors from the social vulnerability literature capture dimensions of SDOH that are of particular significance to health and relatively understudied. Studies show car owners have better overall health as compared to non-car owners [14]. Access to a car can be critical for maintaining employment and educational opportunities, healthcare services, and healthy foods. Mobile homes influence health through multiple social mechanisms such as indoor housing risks (e.g. fire, injuries, toxins), unstable housing tenure, and the characteristics of the surrounding neighborhood [15, 16]. Households with a larger number of occupants tend to have limited income to spend on housing, low-wage employment opportunities, and higher stress levels [17]. Persons receiving social security benefits as the sole source of income experience greater financial and social instability. Female headed households, and/or single mothers have higher poverty levels, constrained socio-economic mobility, and face gender discrimination in the form of lower salaries and employment opportunities afforded to them [18, 19].

This present study is conducted in concert with the Florida Department of Health and the Center for Disease Control and Prevention (CDC), and CTSE’s (Council of Territorial and State Epidemiologists) Sub-County Assessment of Life Expectancy (SCALE) initiative [20]. Identifying the drivers behind differences in LE can provide researchers with the tools to monitor and address the underlying conditions contributing to unequitable health outcomes [21].

The present study draws on both the SDOH and social vulnerability literature to identify relevant factors affecting LE. The study considered well known SDOH such as income, education, race, sex, and urban versus rural differences as well as more specific vulnerability indicators to investigate health disparities. We suspected that neighborhoods with low unemployment, poverty, Native American and/or African American populations, uninsured, and high education levels to have greater average LE. The objective of this study was to assess whether social vulnerability indicators (SOVI) account for additional spatial variation in Florida, U.S. census tract LE estimates over 2009–2013. As an ecologic study, the comparison focuses on population level LE between groups, not individuals.

## Methods

### Study area

Florida is an ideal study area for this analysis due to the state having a large ageing population and geographically varying racial and ethnic compositions. Florida is also the third most populous state in United States and its citizens have drastically different income levels and degrees of socioeconomic position. Only one study examined LE in Florida across census tracts in Nassau County [22].

### Mortality records

Mortality data were obtained from the Florida Department of Health, Bureau of Vital Statistics. Deaths from any cause among Florida residents were included, and geocoded based on residential address. The mean accuracy associated with geocoding residents’ deaths was 97.51% for 2009–2013. According to Florida Department of Health Bureau of Vital Statistics,^1^ accuracy is defined as a percentage of the number of deaths that are successfully geocoded to a census tract divided by the number of residents that year. The Florida Department of Health Bureau of Vital Statistics considers a decedent’s address geocoded if it geographically aligns with the tops of a building or a mailbox. The geocoding process first geocodes the decedent’s address to a high-quality street road network. Unmatched addresses are manually reviewed for errors and put through a second round of geocoding. Death records that were not accurately geocoded were excluded from the calculation of life expectancy. Each death record contained the year of death, state/county/ZIP code/census tract of residence, sex, race/ethnicity, and age at death, broken down into 19 age groups (< 1, 1–4, 5–9, 10–14, 15–19, 20–24, 25–29, 30–34, 35–39, 40–44, 45–49, 50–54, 55–59, 60–64, 65–69, 70–74, 75–79, 80–84, 85+). We included all deaths that occurred between 2009 and 2013. This range was chosen for two reasons: 1) it was the most recent data available at the time of calculation and 2) it covered the year 2010 with the most accurate population denominator data.

#### Population data

Population data were obtained from the 2010 US Decennial Census for the geographies of interest for each of the 19 age groups listed above. The decennial census data include more precise census tract level population counts than the American Community Survey. The population for each geography by age group stratification was multiplied by five (five-year study period) to yield the person-time (population-years) denominator for LE calculations. Therefore, the mortality rate estimates were the overall estimates by age group for both sexes.

### Societal determinants of Health

There are a broad range of social characteristics that are known to influence health. Table 1 lists the societal determinants of health commonly used in SDOH and vulnerability studies [10]. The variables representing societal determinants of health listed in Table 1 are drawn from the SOVI, publicly available from NOAA Office for Coastal Management [23]. The University of South Carolina Hazards and Vulnerability Research Institute maintains and regularly updates the SOVI. The SOVI was constructed using data from the US Census Decennial product (2010) and American Community Survey (ACS) from (2006–2010, 23). The data is readily available, free, and well-studied. The SOVI data contains specific social, housing, economic, and demographic LE indicators that may more precisely represent societal processes impacting LE (Supplementary Table 1). Limitations associated with the SOVI are described in the discussion section. Median value of owner-occupied housing units and median gross rent for renter occupied housing units were excluded from this analysis. These indicators had less explicit links to health outcomes in the SDOH literature. Data for persons under 65 years who are uninsured was obtained from the ACS from (2008–2012). Persons uninsured captures societal processes known to impact health outcomes. SDOH included in this research represent varied societal processes that are known to drive health disparities. Furthermore, these risk factors have established links to a range of health outcomes.

**Table 1:** SOVI Indicators & Beta Coefficients from SDEM (Variables Defined in Text and Supplementary Table 1 in Appendix)

SDOH Components	Coefficients	SDEM Model	SDEM Lag
**Life Expectancy (Y)**	**Intercept**	80.75	NA
**Race/Ethnicity**	*QBLACK	0.01*	0.04***
	*QNATAM	−0.24***	−0.16
	*QASIAN	0.11***	−0.01
	*QESL	−0.004	−0.003
	*QHISP	0.06***	0.02**
**Socioeconomic Position**	*PERCAP	0.00003***	−0.00003
	*QED12LES	−0.05***	− 0.05***
	*QNOAUTO	−0.05***	− 0.05**
	*QRICH200K	0.04**	0.03
	*QPOVTY	−0.01*	−0.003
	*QCVLUN	−0.009	− 0.05***
	*QEXTRCT	0 .04**	0.08***
	*QSERV	0.002	0.03*
	*QFEMLBR	0.01*	−0.01
	*QUNINSUR	− 0.02***	−0.001
**Housing Status**	*PPUNIT	−.25	1.37***
	*QRENTER	−0.006	0.0002
	*QMOHO	−0.02***	−0.001
	*QURBAN	0.0008	−0.01***
	*POPDENS	0.0001***	0.0008**
**Household Structure**	*QFAM	0.005*	0.001
	*QFHH	−0.11***	−0.12***
	*QAGEDEP	0.13***	0.06**
**Gender**	*QFEMALE	−0.06***	0.02
**Vulnerable Populations and Miscellaneous**	*QSSBEN	−0.01	−0.03
	*QNRRES	−0.23***	−0.04
**R**^**2**^		0.71	
**Significance (*p*-value)**	* ≤ 0.05	** ≤ 0.01	*** ≤ 0.001

#### Race/ethnicity

LE disparities for different racial/ethnic groups have been investigated quite extensively. Native American and indigenous populations, who are marginalized and segregated experience the largest U.S. disparity in LE [24]. Social inequities that reverberate throughout African American and Native American communities similarly affect other minority groups as well. However, the effects do not always equate to negative health impacts. For example, some researchers found Hispanic communities have lower socioeconomic position but relatively higher LE, terming this trend the Hispanic Paradox [25]. We selected five racial/ethnic structure measures; the percentage of the population who identify as African American (QBLACK), Hispanic (QHISP), Asian (QASIAN), and Native American (QNATAM), and individuals who speak English as their second language (QESL).

#### Socioeconomic position

Numerous studies reported socioeconomic position and income factors that are associated with disparate LE [6, 9, 26]. Recently studied LE gains were relegated to the more privileged and wealthier social groups [27, 28]. Communities with more financial security enjoy longer life expectancies and better physical and mental health outcomes, while communities that experience financial hardship do not fare as well [29]. Though the physiologic link between socioeconomic position and health outcomes is complex and not fully understood, higher levels of chronic stress reduces life expectancy [30]. In 2010, the poverty level threshold for one person was $11,139. For a family of four, the poverty level threshold was $22,314 [31]. We include a variety of measures to assess the impact of income on LE. These include per capita income (PERCAP), households earning $200,000/year or more, proportion of persons living below the poverty level (QPOVTY), and percentage of occupied housing units with no automobile (QNOAUTO).

Education is one of the most important determinants of a person’s health [3, 4]. Educational attainment is predictive of future employment and income, both of which influence place of residence and health care [4]. In contrast to the race and gender LE gaps, educational attainment and LE disparities have widened [32]. Hendi [28] suggests this disparity is partly caused by older adults with lower education levels compared to an increasing proportion of younger adults with higher education levels [28]. Thus, older, marginalized, less educated communities experience greater disparities [28]. Educational attainment was captured through the measure of the population 25+ years with no high school diploma (QED12LES).

Relatedly, employment has a strong relationship to health outcomes. The intersection of employment with education, income, housing, health care, and insurance coverage along with its disproportionate impact on different racial and gender groups has been highlighted in multiple studies [33, 34]. Singh and Siahpush [26] suggest the unemployment rate in a particular place reflects social and economic inequalities and drives disparate longevity rates and risky behaviors [26]. Employment in extraction industries, such as mining or timber harvesting, increases the likelihood of being injured on the job but may vary from place to place [35]. A majority of the extraction industries present in Florida include; farming, fishing, mining, and timber harvesting. Persons employed in low-skilled service occupations such as childcare, customer service, and housekeeping may be susceptible to negative health impacts from low wages and work-related stress. A meta-analysis suggests blue collar women suffer more pain and work-related injuries than blue collar men or white-collar females [34]. Employment was represented by the proportion of people employed in service (QSERV) or extraction jobs (QEXTRCT), females over 16 in the labor force (QFEMLBR), and civilian labor force’s unemployment percentage (QCVLUN).

Health insurance coverage is primarily reliant on employment and income and is a gauge concerning access to healthcare. More frequent checkups and screenings, has been shown to extend LE, through promoting healthy behaviors, reducing preventable deaths, and managing chronic conditions [1]. Factors affecting access to health care include socioeconomic position, race, gender, age, and geographic location. Barriers to care include lack of insurance coverage, limited income and high cost of care, and living far from healthcare services [36]*.* Access to healthcare was measured by the persons uninsured under age 65 (QUNINSUR).

#### Housing status

Over the past two decades, researchers have shown that the negative health impacts associated with poor housing quality and conditions emanate from biological, physical, and chemical hazards [37]. Unaffordable and/or low-quality housing impacts children’s mental health and may increase their likelihood of having high blood pressure, chronic respiratory problems, and increase their susceptibility to infectious disease [38]. Poor housing quality increases the risks of environmental exposures, infections, injuries, and mental health problems [16]. Negative impacts on health outcomes associated with living in a mobile home are as much shaped by geography as by potentially hazardous conditions in the individual units. Zoning regulations, limiting where mobile homes can be placed, pushed trailer parks into suburban and rural areas [15]. This policy increased the distance and lowered access to employment opportunities, health care and services, healthy food options, and public utilities [15, 16]. More apartments are located near city centers; therefore, renters have greater access to public resources and healthier food options. Housing SDOH factors included in our analysis are percentage of renters (QRENTER), percentage of persons living in mobile homes (QMOHO), and persons per household (PPUNIT).

#### Rural versus urban

The National Advisory Committee on Rural Health and Human Services found that SDOH tend to impact rural health outcomes more negatively than urban and suburban residents [5]. Ethnic minority rural populations also exhibit greater negative impacts than their urban counterparts [39]. Similarly, research found rural community health disparities were further amplified in areas far from urban areas (surrounded by rural neighbors) [9]. Measures such as percent of population living in an urban area (QURBAN) and population density (POPDENS) capture the urban-rural divergence. The U.S. census bureau designated urban census tracts as being those with a population greater than 2500 persons in an area [40].

#### Household structure

Family structure and household composition can have both positive and negative impacts on health. Adults members in a stable family household experience emotional and physical health benefits emanating from shared resources, a sense of responsibility and meaning, and a social support network. Children in traditional two-parent households experience better health outcomes, including better mental health than their counterparts in other family types [41, 42]. Female headed households, with no spouse present, are more likely to have limited economic means and are deprived of the benefits to physical and mental health of cohabiting with a partner [43]. Persons over 65 are more likely to have one or more pre-existing health conditions, to be living on a fixed income, and to suffer from limited transportation options thus, leaving this population group more at risk for adverse health effects [44]. This group may also be more reliant on caregivers [39]. Including the proportion of married couple families with own children under 18 (QFAM), proportion of female headed households with no spouse present (QFHH), and age dependent populations (QAGEDEP) (percent population < 18 years or > 65 years) can offer insight into how household structure affects LE.

#### Gender

Over the past century, women have experienced better health outcomes, including LE, than men. Researchers have found, on average, women live about 5–7 years longer than males [45]. Though men enjoy much of the socioeconomic and political power, there is no direct translation to better health outcomes. Around three-quarters of the gap in LE between genders can be explained by deaths from heart disease and lung cancer as well as by traumatic deaths such as suicide and homicide [46]. However, LE gains for men outpaced the growth for women over the past two decades. Potential explanations include gender inequality, gender roles, and the increases in risky behaviors such as rising tobacco consumption and obesity rates in females [25, 45]. To analyze the gender gap in LE rates, our analysis includes percentage of the female population (QFEMALE).

#### Vulnerable populations and miscellaneous

Persons receiving social security benefits is a marker for analyzing health disparities in the elderly and/or disabled population groups with a fixed income. This group is especially susceptible to price fluctuations in basic goods and commodities which can impact their ability to access and pay for medications and preventative care. Factors in our analysis to account for the vulnerable populations and miscellaneous component include nursing home facilities (QNRRES) and percentage of the population receiving social security benefits (QSSBEN).

### Calculating LE

LE at birth is defined as the number of years that a person can expect to live from the time of birth if current age-specific mortality rates for that specific population remain constant over time. The study used the well-established adjusted Chiang II life table method [47–50]. It assumes that deaths are spread evenly throughout each age interval and is able to handle zero deaths in a given age category*.* Smaller population sizes in the oldest age interval can lead to an overestimation of LE. We selected the oldest age category as 85 years or older which provided a more precise LE estimate with the lowest standard error [47–50].

To achieve sufficient sample size in each age interval, mortality data were temporally aggregated into sequential five-year age categories for each census tract. By calculating LE based on 5 years of mortality data, we increased the reliability of the majority of the estimates (based on standard error). Quality control procedures removed 574 census tracts from the analysis that did not meet the following criteria over the five-year study period: a) small number of deaths: < 50 total deaths, b) small population: < 5000 total population-years, c) unreliable estimates: standard error > 2 years mean LE.

**Fig. 1:**
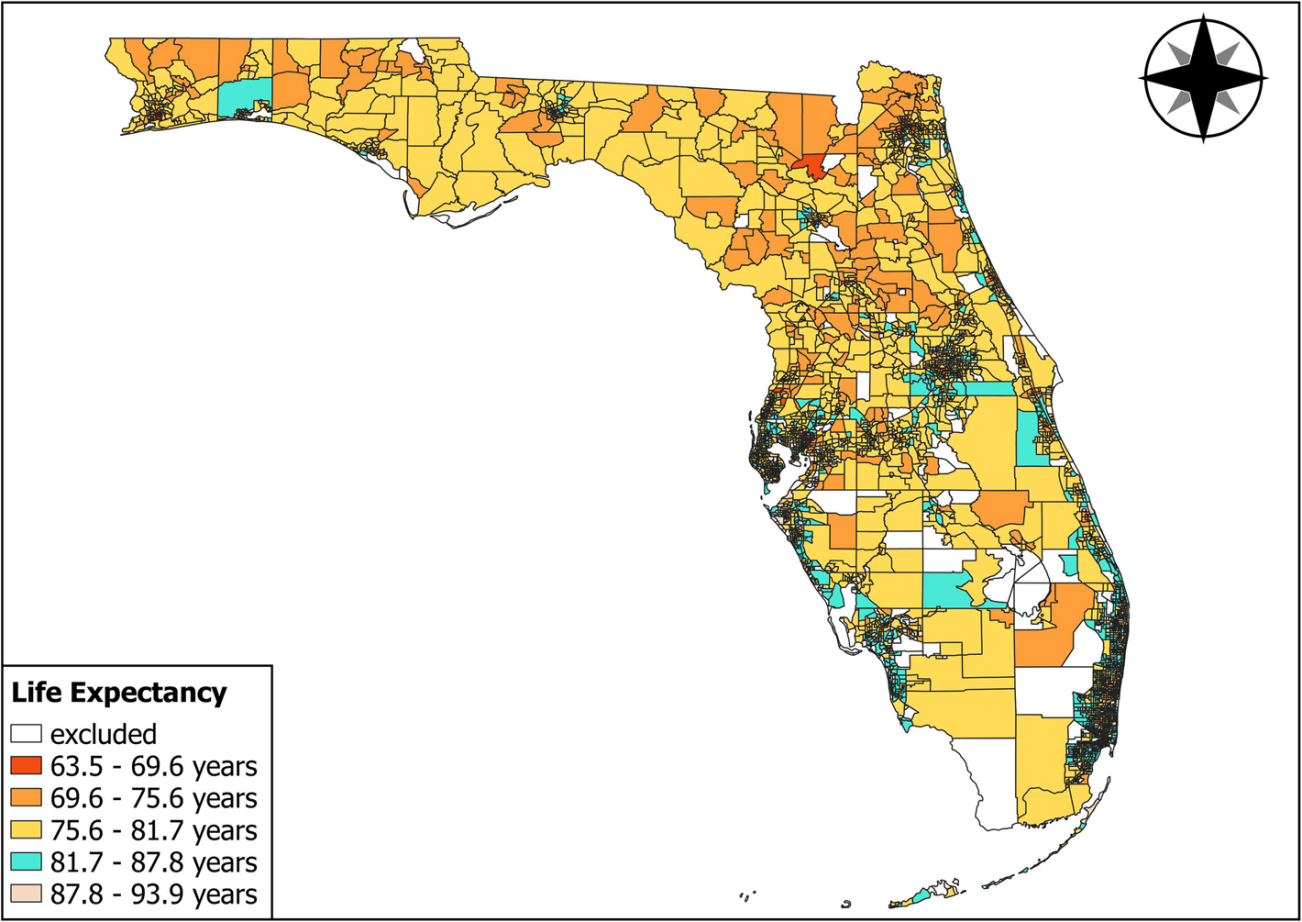
Florida Life Expectancy by Census Tract 2010 (Figure in Color). Map created by the authors using ArcMap 10.4

Census tract LE was calculated using all available deaths over 2009 to 2013 among residents, regardless of age, gender, race or ethnicity. LE estimates were calculated using Excel (Microsoft; Redmond, WA) and SAS 9.3 (SAS Institute; Cary, NC). Data were visualized in ArcMap 10.4 (ESRI; Redlands, CA). Figure 1 shows the spatial distribution of LE by census tract in Florida. LE estimates are relatively homogenous throughout rural census tracts in Florida.

### Data preparation and analysis

To conduct our research, the SDOH and the LE shapefiles were joined by census tract FIPS code using ArcMap 10.4 [51]. The analysis was conducted in the R Statistical Computing and Analysis Program version 3.5.2 [52]. The function errorsarlm in the spatialreg package in R was used to compose the SDEM [53]. The dependent variable was the calculated LE estimate. The VIF (Variance Inflation Factor) tested for multicollinearity between independent variables which may alter the regression beta coefficients and standard errors. The VIF was calculated using the R faraway package. Predictor variables with a VIF over 10, (bivariate Pearson’s Correlation Coefficient > 0.7), were considered for exclusion from the analysis due to multicollinearity [10]. Median age (MEDAGE) and age dependency (QAGEDEP) were correlated (VIF score > 10). We considered proportion of age dependent populations as more representative of vulnerable populations.

#### Statistical model

Conventional ordinary least squares should not be used to analyze neighborhood data which violates the assumption of statistical independence between observations. Essentially, failure to account for non-independence in our data can result in incorrectly calculated regression coefficients [54, 55]. In contrast, spatial models explicitly model non-independence using spatially weighted independent variables and an error term. In other words, a given census tract’s LE estimate is not only related to its own SDOH but to its neighboring tracts’ SDOH as well. The spatially weighted independent variables account for “spatially embedded social processes” and the flow of people, resources, ideas, goods, and services within and between neighboring places [9]. We used the spatial Durbin error model (SDEM) to relate SDOH/vulnerability indicators to LE.

The SDEM models local spatial spillover effects where neighboring SDOH/vulnerability explain a portion of local LE variability. We avoided global spillover models which subjectively restrict the magnitude of spillover effects. We did not consider spatial models that used spatially lagged dependent variables (e.g. spatial Durbin, spatial autoregressive model) which may be more difficult to interpret. The SDEM returns estimates for direct, indirect, and total impacts for each explanatory variable. The direct and indirect impacts or (coefficients) for each of the SDOH factors and their relationship to LE are important for this research. Essentially, the direct impact represents local impacts and the indirect impact represents neighbor impacts. The indirect/neighbor impacts are derived from the relationship of neighboring SDOH values to local LE values. These indirect/neighbor impacts are confined to tracts that share borders. The total impact estimation does not include a measure of significance and is the sum of the direct and indirect effect for each coefficient. In this study, we are interested in identifying local and neighborhood level determinants rather than assessing the total impact of a determinant on LE. Therefore, we decided to not report the total impacts. SDEM builds off of the spatially lagged X model (SLX) to include a spatially lagged error term in addition to spatially lagged and local explanatory variables. The SDEM’s error term accounts for remaining unexplained spatial autocorrelation.

We used a queen’s contiguity spatial weights matrix calculated from the spdep package for input into the SDEM [53]. This neighborhood weights matrix considers all directly adjacent census tracts as neighbors [53, 56]. The spatial weights were row standardized to account for units with different numbers of neighbors. A global Moran’s I test was performed to check for the presence of spatial autocorrelation in the residuals from the SDEM.

## Results

The census tract LE estimate for Florida is 79 years. The minimum LE estimate for a census tract was roughly 63 years [CI: 60.6–66.2] and the maximum was 93 years [CI: 91.1–96.6]. Table 1 reports the beta coefficients and *p*-values (* ≤ 0.05, ** ≤ 0.01, *** ≤ 0.001) for the SDEM statistical analysis. The results are the impact of a particular factor on LE when controlling for the other factors included in the analysis. The direct and indirect effects in the model are the coefficients of the independent and spatially lagged independent variables respectively. Besides population density (POPDENS) and per capita income (PERCAP), each indicator is standardized to represent a proportion of a particular population in a census tract in Florida. This allows for comparisons to be made between beta coefficients for the indicators included in the study. By convention, we used a *p*-value of 0.05 to assess statistical significance of the SDEM. We found our model to be statistically significant (*p*-value < 0.05) for predicting life expectancy with the SDOH included. Model diagnostics were performed to ensure residuals were independent and normally distributed, the residual error term was homoscedastic and relatively constant, and linearity was not violated. Based on the likelihood ratio test and the AIC score, the SDEM best fit the data. Around 71% of the variance in LE, R^2^ = .71, was explained by the SDOH/social vulnerability factors. A global Moran’s I test on the SDEM residuals showed no significant spatial autocorrelation (*p*-value > 0.05). A sensitivity analysis including all 4102 census tracts with LE estimates was performed and yielded relatively consistent results with the reduced model (Supplementary Table 2). Only the spatially lagged indicators, per capita income (PERCAP) and population density (POPDENS) changed in both sign and significance.

The analysis of the racial/ethnic indicators returned significant results and were consistent with the broader literature. The census tracts with higher Native American populations (QNATAM) had notably lower LE. After controlling for all other factors, every 1 % increase in the proportion of Native Americans, decreased LE by 0.24-years (standard error: 0.06, *p*-value < 0.001). The Hispanic Paradox was supported by the results that show a positive relationship between Hispanic population (QHISP) and a 0.06-years (standard error: 0.006, *p*-value < 0.001) increase in LE. Locally, the percentage of the African American population (QBLACK) in a census tract was positively related to LE. A significant spatially lagged effect of 0.04-years (standard error: 0.008, *p*-value < 0.001) increase in LE was measured.

The socioeconomic SDOH and their association to LE supported previous research. Consistently, the income of the richest Americans is associated with an increase in LE. For every percentage increase of households earning more than $200,000/year (QRICH200K), there would be an estimated 0.04-years (standard error: 0.01, *p*-value < 0.01) increase in the LE, locally. Financial constraints and limited mobility reflected through a lack of automobile ownership (QNOAUTO) yielded a significant impact of − 0.05-years (standard error: 0.008, *p*-value < 0.001) decrease in LE. Proportion of persons living below the poverty level (QPOVTY) had a significant impact of − 0.01-years (standard error: 0.007, *p*-value < 0.05) on LE.

Employment, health insurance status, and local labor conditions were significant for predicting LE. Proportion of people employed in extraction jobs (QEXTRCT) was positively associated with LE. An estimated 0.04-years (standard error: 0.01, *p*-value < 0.01) increase in LE would be observed for every 1 % increase in extraction jobs. Locally, the proportion of unemployed persons (QCVLUN) in a census tract was not related to LE, though a significant spatially lagged effect of − 0.05 years (standard error: 0.01, *p*-value < 0.001) decrease in LE was observed. Females in the labor force (QFEMLBR) yielded a significant 0.01-years (standard error: 0.007, *p*-value < 0.05) impact on LE.

Associations between housing characteristics and geographic proximity were assessed in relation to LE. The percentage of persons living in mobile homes (QMOHO) was associated with a − 0.02-years (standard error: 0.003, *p*-value < 0.001) decline in LE. The density of people per housing unit had a significant spatially lagged impact on LE. A spatial lagged effect for persons per unit (PPUNIT) of 1.11-years (standard error: 0.38, *p*-value < 0.01) increase in LE was measured. Tracts with larger population densities (POPDENS), which are more likely to be urban, were predictive of longer LE. This relationship likely accounts for the insignificant local impact of urban areas (QURBAN). While urban areas (QURBAN) had no significant local relationship to LE, a significant negative spatially lagged impact of − 0.01-years (standard error: 0.003, *p*-value < 0.001) to LE was observed.

Each household structure variable included in this analysis was significant for estimating LE. Age dependent populations (QAGEDEP) and proportion of families (QFAM) were both positively and significantly associated with LE. Proportion of families was associated with a local 0.005-years (standard error: 0.002, *p*-value < 0.05) increase in LE. The local impact of age dependent populations was positively associated with 0.13-years (standard error: 0.01, *p*-value < 0.001) increase in LE. The local impact of female headed households (QFHH) was associated with a decrease of − 0.11-years (standard error: 0.01, *p*-value < 0.001) in LE for every 1% increase in the population.

Gender indicators exhibited significant local and neighboring associations to LE. A 1% increase in the proportion of females (QFEMALE) was estimated to produce a negative local impact of − 0.06-years (standard error: 0.01, *p*-value < 0.001) to LE.

Older adults receiving government support may exhibit different life expectancies. A one unit increase in the proportion of nursing home residents (QNRRES) decreased LE by − 0.23-years (standard error: 0.03, *p*-value: 0.001). Persons receiving social security benefits (QSSBEN) did not exhibit a significant relationship to LE.

## Discussion

LE is place-based and must be considered in a local geographic context. We find population level LE is complexly related to multiple factors. This study moves beyond prior research by evaluating the neighbor impact of SDOH on local LE across census tracts in Florida. Our approach accounts for spatially embedded social processes across census tracts. This approach enabled us to identify the significant local and neighbor factors contributing to differences in local LE. Therefore, in small area investigations into differences in LE, it is important to consider spatial relationships between neighborhoods.

Our results shed light on important factors that contribute to differences in life expectancy at the population level in Florida. Notably, the proportion of Native Americans had the largest impact on LE among the racial/ethnic categories. This is consistent with other U.S. and global health disparities and native/indigenous communities’ studies [57]. In the U.S., counties with Native American reservations in North and South Dakota had the lowest LE compared to all other counties in the U.S. [7]. In Canada, indigenous people’s experience around a 12-year gap in LE in comparison to non-indigenous persons [58]. Many of the underlying factors that contribute to health disparities, such as the inequitable distribution of money, resources, and power, have existed since colonization and are still relevant today [57].

The local/neighborhood level may be able to distinguish significant SDOH affecting relationships between LE and race. These relationships are likely to be aggregated over in a county level analysis. For example, the county level relationship between the percentage of African Americans and LE was not significantly related to mortality [25]. The present study found that increasing the proportion of African Americans in the neighboring census tracts positively increased LE. This result aligns with recent research measuring the impact of residential segregation on mortality rates for African American communities. African American communities living in segregated urban areas experience increased longevity and lower mortality rates than African American communities in integrated urban areas [59]. In rural areas, minorities reported their health care needs were met more frequently in segregated rural areas than others living in integrated rural areas [60]. Living in a black enclave may allow communities to pool their social capital and resources to promote positive health impacts.

Similarly, the proportion of Hispanic population in a census tract had a positive relationship with LE. Support for the Hispanic Paradox may be related to the long-term benefits of educational attainment and the types of Hispanic groups living in Florida. Borrell and Lancet [59] observed mortality advantages over non-Hispanic whites varied by nativity status. In particular, their findings suggest Cuban women, 45–64 years old, were around two times less likely to die than non-Hispanic White women [61]. Cubans made up around 28% of the Hispanic population in Florida in 2013 [62]. In comparison to other Hispanic immigrant populations, a higher proportion of Cubans graduate high school and receive a college degree [63]. A higher proportion of immigrants from Central and South America who came to the United States in the 1980’s were from a middle-class background and well educated. This group has been more likely to engage in middle to upper class professions and benefit from a stable income and health care benefits [63]**.**

Proportion of persons unemployed (QCVLUN) and persons working in extractions jobs (QEXTRCT) both had significant impacts on LE. Health benefits associated with extraction jobs may be attributed to employment benefits and higher incomes. Rural communities, where these jobs are often located, may experience positive impacts to local and neighboring LE from increased economic activity [35]. Negative neighborhood impacts from persons unemployed (QCVLUN) to LE may reflect a lack of investment and economic development in disadvantaged communities and health care differences between residents [26]. Tracts surrounded by neighbors with high unemployment rates are likely to have a similar socioeconomic composition. This relationship could be explained by the compounding effects of residential and income segregation by race/ethnicity and/or socioeconomic position and the disparities apparent in educational and employment opportunities in disadvantaged communities.

A positive spillover person per household (PPUNIT) LE effect may reflect geographic dynamics*.* Myers and Baer [64] identified four population groups susceptible to being in an overcrowded household [64]. These are recent immigrants, Asian and Hispanic populations, and lower-income households. The persons per household spillover effect may point to different processes in urban versus rural context. In metropolitan areas, LE is likely to be higher due to the benefit from proximity to resources and health care services. Interestingly, exurban or rural areas adjacent to urban areas may benefit from access to urban amenities. Urban and rural dynamics may also be captured by the negative association between percentage of mobile homes and LE. Rural residents have less access to preventative healthcare services, fresh foods, and employment and educational opportunities [60]. Including an interaction term for urban/rural differences would provide further insight into these LE processes. An interaction term could unveil how SDOH operate separately in rural and urban neighborhoods.

Family structure factors had contrasting local and spatially lagged impacts on LE. Russel et al. [42], suggested family structure is an important social group that is not commonly considered in SDOH research. Single parent and two parent households were differentiated in this analysis through the local and spatially impacts of proportion of families (QFAM), proportion of age dependent population (QAGEDEP), and proportion of female headed households (QFHH). Families and age dependent populations, both had significant positive relationships to LE. Comparatively, female headed households (QFHH) had significant negative local and spatially lagged impacts to LE. Limited economic resources may influence this relationship as single mother households are considered the “poorest of the poor” [18]. Social support systems, multiple incomes, and psychological benefits associated with a stable household may in part explain this observed relationship [42]. Evidence from our results indicate that family structure may play a role in promoting healthy behaviors and better health outcomes.

There are several limitations we recognized when conducting this study. First, it is important to note that analyzing neighborhood-level SDOH reveals how drivers of disparate health outcomes operate at the local and neighborhood level but does not establish causation between LE disparities and SDOH [9, 65]*.* Second, ecologic studies are limited for inferring causation. Therefore, results from this study are subject to the ecologic fallacy and are limited to relationships or associations between dependent variables and explanatory variables and do not necessarily hold true at the individual level. Essentially, results for the factors affecting life expectancy cannot be reduced to the individual level, but rather reflect the health disparities related to specific populations groups. Third, these findings are subject to the modifiable areal unit problem and should not be generalized to other spatial scales of analysis. Fourth, the dimensions of SDOH and social vulnerability indicators may not capture the complex interplay of SDOH processes. It is plausible that factors not captured by existing data sources may reflect important LE processes. Fifth, a limitation in this study involves the calculation of LE. The LE estimates do not consider internal migration and do not necessarily capture the health of population living in an area for their entire lifetime. Caution should be exercised in interpreting the LE estimates as reflective of more or less healthy places, but rather should be considered as representative of the place-specific health of a population at a certain point in time. For example, areas in southern & central Florida with a high proportion of retirees from other states and/or part time residents may have less reliable metrics. Similarly, the total number of deaths over a 5-year period may be relatively small. Therefore, this dataset, analysis, and results contains more uncertainty than a county level analysis. Mass casualty incidents could have an outsized influence on the neighborhood level death counts and the resulting LE estimate. Sixth, under-registration is an important limitation to consider in this study. Under-registration can increase the uncertainty associated with LE estimates. In particular, undocumented migrants’ deaths may not be captured in the LE estimates which can lead to uncertainty in the denominator data used in the LE calculations. Deaths included in the LE calculations had an indication on the death certificate that the decedent was a Florida resident. Similarly, small variation exists in the completeness of death registration. Although this variance in death registration cannot explain the total variability, it contributes to the variation in LE estimates. This can lead to an underestimation of spatial health disparities [7]. Novel methods not explored in this study exist which could improve the robustness of LE estimates for small geographic areas [66]. Seventh, the average values of LE for the years 2009–2013 temporally overlap with the SOVI from 2010, but are offset. Macro-level events such as the great recession and the opioid epidemic could have potentially impacted the LE estimates and/or census data. Eighth, some limitations exist with the SOVI; mainly in regards to census data having different levels of uncertainty. Finally, local area variation in LE may be influenced by physical and environmental characteristics of an area, which may better reflect and describe the health disparities associated with living in a particular place. Rather, relationships point toward impacts between factors included in our study and LE and are suggestive of the societal processes driving disparities.

Considering social vulnerability indicators in addition to SDOH metrics allowed us to more accurately characterize and investigate spatial health disparities. Specifically, indicators related to housing status and structure were unique in this line of research and had an unexpectedly large impact on LE. The neighbor impact of persons per household (PPUNIT) was useful for parsing out rural-urban differences in LE. Comparatively, the proportion of mobile homes (QMOHO) captured the interplay of the negative health impacts associated with neighborhood characteristics, living in a rural area, and indoor housing risks. Positive impacts to LE associated with age dependent populations (QAGEDEP) and proportion of married couple families with own children (QFAM) indicates families may promote healthy behaviors and social cohesion within communities. An intriguing contrast to this relationship can be observed through the negative impact female-headed households, with no spouse present, (QFHH) had on LE. Results from the SDEM for associations between the SDOH and LE indicate that the SOVI dataset is applicable to future studies aimed at investigating spatial health disparities in Florida and the southeastern United States. Introducing these factors into future analysis would be useful for describing how urban-rural differences, housing conditions, and family structures produce disparities.

## Conclusions

We identified important determinants associated with LE and attempted to interpret how underlying social mechanisms and spatial processes explain the variation in LE. This framework can be used to investigate geographic disparities in health outcomes through a SDOH approach at the local level. Using the census tract level spatial structure to measure local and neighborhood SDOH, we successfully explained a large portion of the variation in LE. By combining commonly used SDOH and social vulnerability risk factors, we were able to more precisely characterize health disparities. Identifying important factors affecting life expectancy can play a role in improving public health strategies and targeting health interventions to reduce local health disparities.

## Supplementary information

**Additional file 1: Table S1.** Description of the 26 SOVI Indicators.

**Additional file 2: Table S2.** Sensitivity Analysis Results 4102 Census Tracts Included.

## Data Availability

Data and Rcode that supports the findings of this study are available from https://github.com/blm17e/Health-Geography. The original data is available from Florida Department of Health (http://www.flhealthcharts.com/ChartsReports/rdPage.aspx?rdReport=ChartsProfiles.LifeExpectancyProfile) and publicly available from NOAA Office of Coastal Management (https://coast.noaa.gov/digitalcoast/data/).

## Abbreviations

SDOH: Social determinants of health
LE: Life expectancy
SDEM: Spatial Durbin error model
SOVI: Social Vulnerability Index
CDC: Center for Disease Control and Prevention
CTSE: Council of Territorial and State Epidemiologists
SCALE: Sub-County Assessment of Life Expectancy
ACS: American Community Survey
VIF: Variance inflation factor
SLX: Spatially lagged X model

## Footnotes

1 Per correspondence with Karen Freeman (karen.freeman@flhealth.gov) and Katherine McDaniel (katherine.mcdaniel@flhealth.gov) at the Florida Department of Health
