# Supplementary material for: Florida neighborhood analysis of social determinants and their relationship to life expectancy
Source: BMC Public Health. 2020 May 6;20:632. doi: 10.1186/s12889-020-08754-x (PMC7204051; doi:10.1186/s12889-020-08754-x)
Supplement: Supplementary file 1 — Additional file 1: Table S1. Description of the 26 SOVI Indicators. [file 12889_2020_8754_MOESM1_ESM.docx]

| **SDOH Components** | **SOVI Indicators** | **Description** |
| --- | --- | --- |
| **Race/Ethnicity** | *QBLACK | % African American |
|  | *QNATAM | % Native American |
|  | *QASIAN | % Asian or Pacific Islander |
|  | *QESL | % ESL (poorly or not at all) |
|  | *QHISP | % Hispanic |
| **Socioeconomic Position** | *PERCAP | Per Capita Income |
|  | *QED12LES | % Population 25 years or older with no high school diploma |
|  | *QNOAUTO | % Occupied housing units with no automobile |
|  | *QRICH200K | % Households earning mor than $200,000 |
|  | *QPOVTY | % Civilian noninstitutionalized population living below the poverty level |
|  | *QCVLUN | % Population of workforce age (16-64 years) who did not work in the past 12 months |
|  | *QEXTRCT | % Civilian employed population 16 and older employed in farming, fishing, mining, and forestry occupations |
|  | *QSERV | % Civilian employed population 16 and older employed in service occupations |
|  | *QFEMLBR | % Total civilian employed population 16 and older who are female |
|  | *QUNINSUR | % Uninsured total civilian noninstitutionalized population |
| **Housing Status** | *PPUNIT | Average number of people per occupied household |
|  | *QRENTER | % Housing units that are renter occupied |
|  | *QMOHO | % Housing units that are mobile homes |
|  | *QURBAN | % Population living in urban block groups |
|  | *POPDENS | Number of people per square mile land area |
| **Household Structure** | *QFAM | % Own children under 18 living in married couple families |
|  | *QFHH | % Households that are female headed, with no spouse present |
|  | *QAGEDEP | % Population either under 5 yrs of age or 65 or over 2010 (i.e., elderly and young children as a percent of the total population) |
| **Gender** | *QFEMALE | % Population who are female |
| **Vulnerable Populations and Miscellaneous** | *QSSBEN | % Households collecting social security benefits |
|  | *QNRRES | % Population who are 65 and over in nursing facilities |

**Supplementary Table 1:** Description of the 26 SOVI Indicators
