# Supplementary material for: Florida neighborhood analysis of social determinants and their relationship to life expectancy
Source: BMC Public Health. 2020 May 6;20:632. doi: 10.1186/s12889-020-08754-x (PMC7204051; doi:10.1186/s12889-020-08754-x)
Supplement: Supplementary file 2 — Additional file 2: Table S2. Sensitivity Analysis Results 4102 Census Tracts Included. [file 12889_2020_8754_MOESM2_ESM.docx]

| **SDOH Components** | **Coefficients** | **SDEM Model** | **SDEM Lag** |
| --- | --- | --- | --- |
| **Life Expectancy (Y)** | **Intercept** | 74.32 | NA |
| **Race/Ethnicity** | *QBLACK | 0.02** | 0.04*** |
|  | *QNATAM | -0.32*** | -0.17 |
|  | *QASIAN | 0.1** | 0.01 |
|  | *QESL | -0.002 | 0.007 |
|  | *QHISP | 0.08*** | 0.001 |
| **Socioeconomic Position** | *PERCAP | 0.00004 | 0.00007*** |
|  | *QED12LES | -0.07*** | -0.03 |
|  | *QNOAUTO | -0.04*** | -0.05* |
|  | *QRICH200K | 0.09*** | -0.08* |
|  | *QPOVTY | -0.01 | 0.01 |
|  | *QCVLUN | -0.03*** | -0.04** |
|  | *QEXTRCT | 0 .04** | 0.04 |
|  | *QSERV | -0.0005 | 0.03 |
|  | *QFEMLBR | 0.01 | -0.02 |
|  | *QUNINSUR | -0.006 | 0.009 |
| **Housing Status** | *PPUNIT | -1.17*** | 1.65*** |
|  | *QRENTER | -0.01** | 0.01 |
|  | *QMOHO | -0.02*** | 0.01 |
|  | *QURBAN | 0.003 | -0.008* |
|  | *POPDENS | 0.0001*** | -0.0001 |
| **Household Structure** | *QFAM | 0.02*** | 0.02*** |
|  | *QFHH | -0.09*** | -0.09** |
|  | *QAGEDEP | 0.1*** | 0.05* |
| **Gender** | *QFEMALE | -0.08*** | 0.05 |
| **Vulnerable Populations and Miscellaneous** | *QSSBEN | -0.01 | 0.001 |
|  | *QNRRES | -0.2*** | -0.2* |
| **R²** |  | 0.58 |  |
| **Significance (p-value)** | *≤0.05 | **≤0.01 | ***≤0.001 |

**Supplementary Table 2:** Sensitivity Analysis Results 4,102 Census Tracts Included
